# Central retinal artery occlusion associated with persistent truncus arteriosus and single atrium: a case report

**DOI:** 10.1186/s12886-015-0126-8

**Published:** 2015-10-19

**Authors:** Cheng-wei Lu, Jun Wang, Dan-dan Zhou, Ji-long Hao, Ling-ling Liang, Xiao-hong Li, Peng Hui

**Affiliations:** Department of Ophthalmology, the First Hospital of Jilin University, No. 71 of xinmin St, Changchun, Jilin Province 130021 China; Department of Ultrasound, the First Hospital of Jilin University, No. 71 of xinmin St, Changchun, Jilin Province 130021 China; Department of Radiology, the First Hospital of Jilin University, No. 71 of xinmin St, Changchun, Jilin Province 130021 China

**Keywords:** Central retinal artery occlusion, Persistent truncus arteriosus, Single atrium

## Abstract

**Background:**

Central retinal artery occlusion (CRAO) is an ocular emergency and most of the cases present with painless sudden persistent loss of vision in the range of counting fingers to perception of light. The presentation of CRAO is associated with a variety of medical conditions. We report a rare case of CRAO associated with persistent truncus arteriosus (PTA) and single atrium in a female patient.

**Case presentation:**

A 23-year-old woman was admitted due to sudden painless visual loss in the left eye. On examination visual acuity of light-perception was noted in the left eye with a left relative afferent pupillary defect. Fundoscopic examination revealed retinal ischemic whitening, constriction of the arteriole and venule with segmentation and typical “cherry-red spot” suggesting CRAO. The patient was treated with ocular massage and anterior chamber paracentesis. She was commenced on 150 mg of aspirin and also received hyperbaric oxygen therapy. An echocardiogram revealed PTA and single atrium. A diagnosis of CRAO associated with PTA and single atrium was made.

**Conclusion:**

The ophthalmologist should enquire about congenital and acquired cardiac abnormalities in patients with CRAO and consider such abnormalities to be possible sources of emboli.

## Background

Persistent truncus arteriosus (PTA) and single atrium are both rare congenital cardiac syndromes, and their occurrence together is extremely rare. PTA accounts for under 1% of all congenital heart diseases, and over 80% of patients succumb to heart failure in infancy [1]. Central retinal artery occlusion (CRAO) is an ocular emergency, and the etiology of it is usually associated with atherosclerotic risk factors and the presence of intravascular (carotid artery, aortic arch) or intracardiac embolic material. At young age, CRAO may be a manifestation of inherited or acquired thrombophilia [2]. However, CRAO associated with congenital cardiac anomaly is uncommon. According to a literature review, a CRAO case with PTA and single atrium has not been described previously. Herein we first report a rare case of CRAO in a Chinese female patient who had both PTA and single atrium with survival to age 23.

## Case presentation

**Fig. 1:**
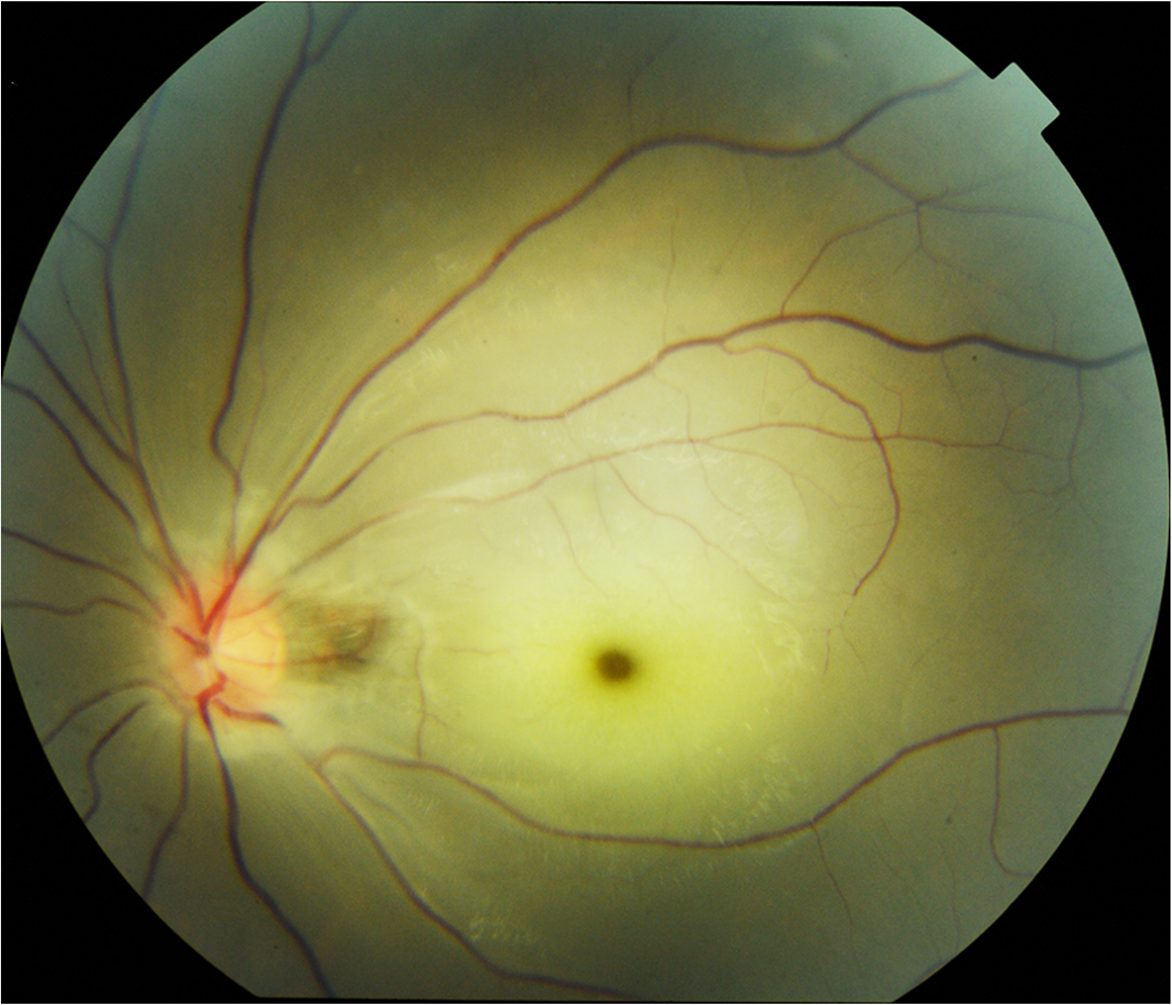
Fundus photography of the affected eye. Fundus examination revealed retinal chemic whitening, and a cherry-red spot in the foveal area

**Fig. 2:**
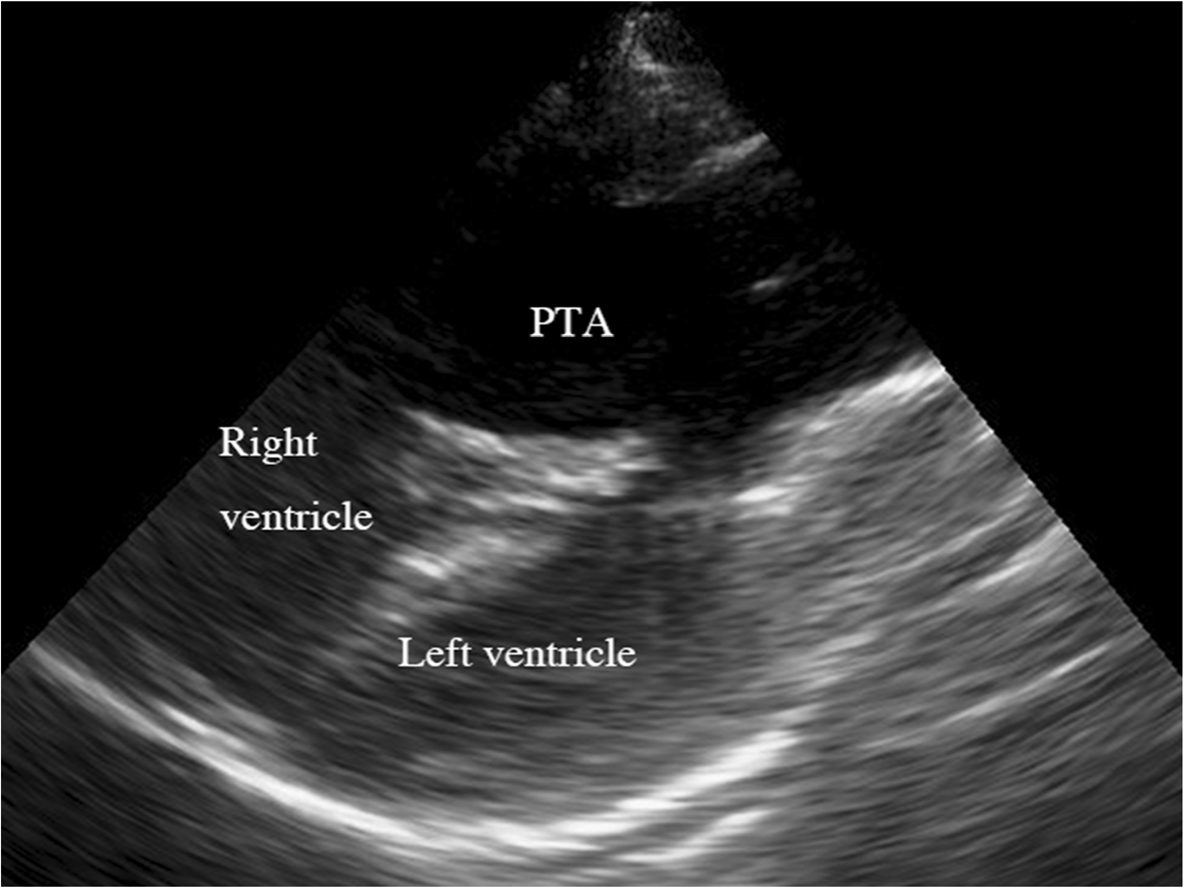
The image of echocardiogram. An echocardiogram revealed PTA overriding both ventricles

**Fig. 3:**
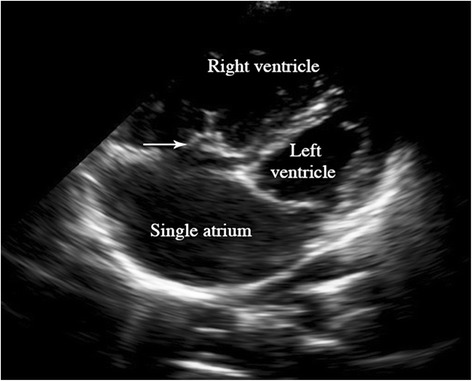
Echocardiographic image. An echocardiogram revealed single atrium and tricuspid valve prolapse (*arrow*)

A 23-year-old woman presented with sudden painless visual loss in the left eye of 100 min. She had been diagnosed with PTA at the age of 1 month. In medical history, she had no ophthalmic problem and had maintained good visual acuity. There was no history of vascular occlusion affecting other organs. On examination visual acuity of 20/20 in the right and light-perception was noted in the left eye with a left relative afferent pupillary defect. Anterior segment examination was unremarkable with normal intraocular pressures. Fundoscopic examination revealed retinal ischemic whitening, constriction of the arteriole and venule with segmentation and typical “cherry-red spot” suggesting CRAO (Fig. 1). The patient was treated with ocular massage and anterior chamber paracentesis. She was commenced on 150 mg of aspirin and also received hyperbaric oxygen therapy. Her visual acuity returned to hand motion in the temporal direction. Fluorescein angiography of the right eye carried out on the second day was normal, whereas the left eye showed a delay in arterial filling and large areas of nonperfusion. An echocardiogram was performed (Figs. 2 and 3) and revealed a single and dilated truncal artery overriding both ventricles, a subtruncal ventricular septal defect (VSD) and absence of pulmonary valve and arteries. A diagnosis of persistent truncus arteriosus was established. The annulus size of common truncus was 41 mm. A single atrium, normal function of the left ventricle, and a hypertrophied right ventricle with severe tricuspid regurgitation were shown. Ultrasonography of the carotid arteries was unremarkable. Further evaluation for an underlying cause was unrevealing, with a normal erythrocyte sedimentation rate, C-reactive protein level, blood count, renal function and ionogram, liver tests, glycosylated hemoglobin and hemostasis. A head computed tomography scan at this time demonstrated no abnormalities.

## Discussion

CRAO is an ocular emergency and the incidence is estimated to be 1 in 100,000 people [2]. In 1859, von Graefes [3] first described CRAO as an embolic event to the central retina artery in a patient with endocarditis. Most of the cases present with painless sudden persistent loss of vision in the range of counting fingers to perception of light. Anterior segment evaluation is usually normal except for the presence of an afferent pupillary defect. Initially, fundus may appear relatively normal. Eventually, hypoxia results in ischemic whitening of the retina, most pronounced at the posterior pole. A cherry red spot is typical and found in about 90 % of cases.

The presentation of CRAO in a young individual is associated with a variety of medical conditions, including hyperhomocysteinemia, temporal arteritis, systemic lupus erythematosus, sickle cell disease, platelet aggregation abnormalities, and migraine. But Cardiogenic emboli are a very rare cause of CRAO. While atrial fibrillation and left ventricular dysfunction were shown to be the most common cardiac sources, extracardiac sources (mainly aortic and carotid plaques) are associated with CRAO in the large majority of patients [4]. Unfortunately, the source of embolism remains unclear in about 45 % of the patients [5]. Other proposed mechanisms include infective endocarditis, left atrial thrombosis and myxoma, aortic arch atheroma, mitral annulus calcification, left atrial appendage thrombus, valvular abnormalities, papillary fibroelastoma, and patent foramen ovale [6]. In the present cases, no other source of embolism could be found except for the described exceedingly rare congenital heart disease. However, association of CRAO with PTA and single atrium, to our knowledge, has not been reported.

**Fig. 4:**
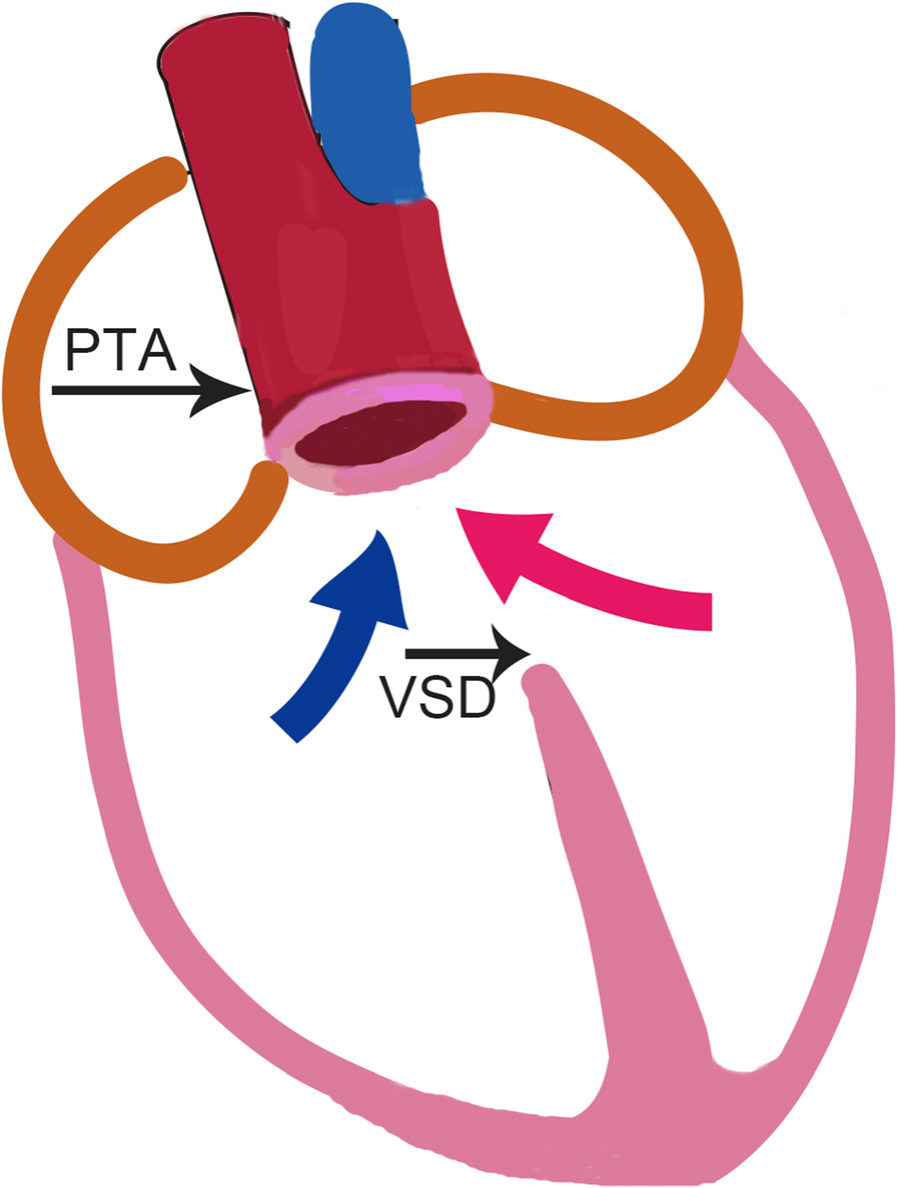
A diagram illustrating the nature of PTA. PTA (*black long arrow*) overrided both ventricles and received blood flow from both ventricles (*red and blue arrows*). Pulmonary artery arose from the PTA and VSD (black short arrow) was present

We first present a patient with CRAO who was born with the simultaneous occurrence of two congenital cardiac defects, which are individually uncommon. PTA is usually termed as a single great artery arising from the base of the heart that supplies systemic, coronary and pulmonary blood flow (Fig. 4). Almost all cases are associated with VSD. The natural history of PTA is poor. Although this patient did not have well-developed pulmonary arteries (PAs), sufficient circulation to the PAs by means of collateral vessels and balanced systemic circulation may have been present. This probably resulted from protection of the pulmonary circulation induced by the brachial artery or hypoplastic branch PAs in this PTA patient. Single atrium is a failure of development of the embryologic components that contribute to the atrial septal complex. Both PTA and single atrium can lead to lower-than-normal oxygen levels in the arterial blood, pulmonary hypertension, and secondary tricuspid valve prolapse.

Therapy for CRAO remains highly uncertain and controversial. It is thought that removal of the embolus within 90 min gives the best chance at recovery, but irreversible damage occurs after 4 h [7]. Treatment measures include increasing blood oxygen content and dilation of retinal arteries, reducing intraocular pressure immediately through medications (acetazolamide 500 mg i.v or orally, topical beta blocker), ocular massage, and anterior chamber paracentesis, and the use of thrombolytics [8]. Hayreh and Zimmerman [9] described that visual outcomes differed between types of CRAO. Vision improves in 22 % of eyes with nonarteritic CRAO, whereas it improves in 67 % of eyes with nonarteritic CRAO with cilioretinal artery sparing. In the present case of nonarteritic CRAO including cilioretinal artery, visual improvement appeared to be poor. These findings suggested that earlier intervention is necessary for the management of CRAO to preserve visual function.

## Conclusion

In summary, we first describe an extremely rare case of CRAO associated with PTA and single atrium. When we encounter acute painless visual impairment, CRAO should be considered and treated as soon as possible, and the ophthalmologist should enquire about congenital and acquired cardiac abnormalities in patients with CRAO and consider such abnormalities to be possible sources of emboli.

## Consent

Written informed consent was obtained from the patient for publication of this case report and any accompanying images. A copy of the written consent is available for review by the Editor of this journal.

## Abbreviations

CRAO: Central retinal artery occlusion
PTA: Persistent truncus arteriosus
PAs: Pulmonary arteries
VSD: Ventricular septal defect
